# Addiction by Any Other Name is Still Addiction: Embracing Molecular Neurogenetic/Epigenetic Basis of Reward Deficiency

**Published:** 2020-01-01

**Authors:** Drew Edwards, A. Kenison Roy, Brent Boyett, Rajendra D. Badgaiyan, Panayotis K. Thanos, David Baron, Mary Hauser, Sampada Badgaiyan, Raymond Brewer, David B. Siwicki, William Downs, David E. Smith, Kenneth Blum

**Affiliations:** 1 Drew Edwards & Associates, Lakeview, FL, USA; 2 Department of Psychiatry, Tulane University School of Medicine, New Orleans, LA, USA; 3 Division of Neuroscience & Addiction Therapy Research, Pathway Healthcare, LLC., Birmingham, AL, USA; 4 Department of Psychiatry, Icahn School of Medicine Mt Sinai, New York, NY, USA; 5 Department of Psychiatry, South Texas Veteran Health Care System, Audie L. Murphy Memorial VA Hospital, San Antonio, TX, USA; 6 Long School of Medicine, University of Texas Medical Center, San Antonio, TX, USA; 7 Department of Psychology & Behavioral Neuropharmacology and Neuroimaging Laboratory on Addictions (BNNLA), Research Institute on Addictions, University at Buffalo, Buffalo, NY, USA; 8 Western University Health Science Centers, Pompano, CA, USA; 9 Division of Addiction Services, Dominion Diagnostics, North Kingston, RI, USA; 10 Department of Nutrigenomics, Geneus Health, LLC, San Antonio, TX, USA; 11 Division of Nutrigenomics, Victory Nutrition International, LLC., Lederoch, PA, ISA; 12 Haight Ashbury Free Clinics, San Francisco, CA, USA; 13 Department of Psychiatry, University of Vermont, Burlington, VT, USA; 14 Eotvos Loránd University, Institute of Psychology, Budapest, Hungary; 15 Department of Psychiatry, Wright University Boonshoft School of Medicine, Dayton, OH, USA

The Human Genome Project and the database it created established a plausible observatory, so to speak, for scientists to identify the etiology of genetic variants and their expression. It is well-known that “Single Nucleotide Polymorphisms” (SNPs) which involve the cumulative presence of nucleic acids in sufficient volume and proximity along the DNA strands to create novel variants in the transcription and encoding of replicate genes--thus creating phenotypical risk for pathological expression [1]. One of these created phenotypes involves the molecular rearrangement of known base pairs sitting in chromosomes leading to an uncontrollable desire to self-administrate various drugs and even risky behaviours to overcome a known neurochemical deficiency or hypodopaminergia resulting in drug or non-drug seeking behaviours [2].

In the field of *behavioral and addiction medicine researchers* [3-6] have identified numerous SNPs and genetic variants in several candidate genes. For example, CADM2, is associated with sensation seeking and drug experimentation. CADM2 is just one of many candidate genes associated with Substance Use Disorder [7]. However, following the seminal work of our group on the first association of the DRD2 A1 allele and severe alcoholism the field of Psychiatric Genetics was born. A PUBMED search (12-6-19) reveals that there are now over 22,981. Along these lines is it well known that there over 393 genes that have associated with drug and alcohol seeking behaviours, whereby the two major pathways that have been consistently identified are glutaminergic and dopaminergic [8] While there have been many reports trying to untangle the specific role of dopamine in reward processing, the idea of “liking” and “wanting” revealed that in terms of dopaminergic mechanisms “wanting” seems to be the most relevant [9, 10]. However, it is well-established that dopamine especially in the brain reward circuitry is responsible in-part for motivation, cognitive abilities, achievement of pleasure, pain tolerance and even anti-stress functions [11]. One important aspect that requires consideration in terms of both treatment and prophylaxis of addictive behavioral seeking is balancing the Brain Reward Cascade (BRC) with the net effect of ensuring “dopamine homeostasis”. Failure to do so will result in high relapse rates [12]. One major issue that we take issue with has to do with the long term implications of treating opioid addiction with agonistic (methadone) or even partial agonistic opioids (buprenorphine) or even blocking opioid receptors with injectable Naltrexone [13]. In spite of the positive life saving aspects of using MAT to treat opioids and even alcohol, especially linked to reducing “societal harm” there is benefit in terms of quality of life especially in terms of prevention of overdose. However, while on these pharmaceuticals long term, they could impair cognitive [14]. In fact, Hill et al., [15] evaluating emotional reactivity as measured by automatic detection of speech, found that long-term combinations of buprenorphine and naloxone resulted in a flattening of affect among some patients, compared to the general population and early attenders of Alcoholics Anomalous groups (p< 0.01). From as early as the late 60’s notable work from Myers group [16] showing the role of serotonin in alcohol intake and the initial work of Blum’s group [17] showing the blocking of ethanol dependence with the narcotic antagonist naloxone, and Davis’s group [18] showing the involvement of isoquinolones (an opioid like condensation product of dopamine and acetaldehyde among others) initiated the concept of common mechanisms for opioids and alcohol [19]. This early work provided the actual framework for Blum’s original concept he termed Reward Deficiency Syndrome (RDS) [20]. Following many years of study globally with 185 PubMed listed articles, RDS is featured as an abnormal psychological disorder in Sage Encyclopaedia of Clinical and abnormal Psychology [21].

These findings are supported through transcriptome analysis (the volume of messenger RNA molecules). Addictive disease (drug and non- drug (process) [22], depression [23], anxiety disorder [24], attention-deficit/hyperactivity disorder (ADHD) [25] and post-traumatic stress disorder (PTSD) [26] are all phenotypical conditions. Because these conditions share candidate genes and the co- occurring expression of neuropsychiatric conditions affecting the midbrain, ventral striatum, the term Reward Deficiency Syndrome has been coined to describe their shared etiology and pathophysiology [27].

In addition, new imaging technology has shed much needed light on the brain’s “functional anatomy.” Over the last five decades of research concerned with the role and significance of specific neurotransmitters, their bioavailability, and the neurocircuitry that enables the brain to communicate electrochemically, has framed our modern-day view of all addictive behaviours [28]. Pleasure, contentment, mood, focus and cognition all conspire to determine our mental and behavioral health, our life trajectory, and quality. For those with Addictive Disease, and its most common comorbidities, life can seem empty and hopeless.

It’s important to note that SNPs and phenotypical risk factors are not in themselves causal. Environmental and familial stressors combined with genomic variants may result in a disease or condition being expressed. Over the last two decades our new understanding of the role environmental factors play in terms of gene expression termed epigenetics, has paved the way to understanding the simple well-known equation P = G + E. Where P = Addiction Phenotype; G = Genetic Trait; E = Epigenetic impact which could occur without changing DNA per se [29]. One strong example of the role of epigenetics as studied by Szutorisz et al. [30] whereby they found that parental THC exposure leads to compulsive heroin-seeking and altered striatal synaptic plasticity in the subsequent generation. Thus based on this and other definitive work [31], we can now ascribe a better understanding of why persons without genetic risk factors who persistently use intoxicants, or experience trauma or chronic stressors can cause epigenetic changes that redefine pleasure and reward through neuroadaptation for up to at least subsequent generations.

Finally, the good news is that genetic testing technology currently exists which can identify SNPs and determine one’s specific risk for phenotypical Reward Deficiencies. This test is known as the Genetic Addiction Risk Score (GARS) coupled with a precision matched Pro-dopamine regulator (KB220) and this system has been referred to as Precision Behavioral Management (PBM) [32, 33]. Who knows, perhaps one day CRISPR or other gene editing technologies (gene splicing, editing) will prevent the expression of some phenotypical risks for addictive disease. This in-part could be accomplished for example by editing the DNA code to change the mRNA expression of DRD2 A1 carriers to expressing the so called normal variant A2 and as such attenuation of self- medicating for a “dopamine fix”.

However, we and most experts agree these newer concepts will not end addiction, as humans are hedonically inclined and, likely, will continue seeking more diverse pleasures, convenience and shortcuts toward reward attainment. In support of this statement, based on the now thousands of subjects GARS tested in the America, it has been found (unpublished) that there is a very high genetic risk for RDS.

But, for now, treatment for addictive disease is most effective within a chronic disease framework, multimodal, of optimal duration and intensity, provided by a highly trained and experienced multidisciplinary team, patient and family cantered, and highly collaborative. We believe with the further incorporation of GARS testing, or other genetically based accurate testing, and having as a goal “dopamine homeostasis” that the trajectory of success and quality of life will be improved [34].

Genetic and epigenetic profiles that underlie disease manifestation, treatment progression, and individualizing treatment need to be better understood in order to advance substance use disorder treatment and patient recovery. Additionally, there needs to be an expansion of comprehensive SUD treatment that is driven by a bio-psycho-social model of treatment. This is outlined in Gustin et al., [35]. Additionally, an operational continuum of care needs to be constructed throughout our healthcare system, so that patients at risk for SUD can be reliably identified and filtered into long-term, continuous treatment, including the use of pharmacies as suggested by Shonesy et al. [36]. Moreover, there are known issues of ineffective continuums of care in the substance use disorder space and as such there is increasing need for more comprehensive, continuous care and not purely MAT medical management. It is known that medical management alone is oftentimes insufficient when dealing with a complex behavioural/psychiatric disease and there are not enough treatment providers that safely integrate medication into comprehensive substance use disorder treatment [35]. Finally, it is also known that MAT, important for harm reduction [37], may result in cognitive impairment as observed by Hill et al. using a true ground lie detector [15].

While it is known that throughout the literature there have been many names given to addiction, RDS [38], the other side of darkness [39], anti- reward [40], dopamine deficiency [41], endorphin deficiency [42], our take home message is that Addiction by any other name is still addiction but what one becomes addicted to serves as a modifying nosology [37, 43]. So like a rose as espoused by William Shakespeare, a rose by any other name is still a rose.
